# The Inhibition of Histone Deacetylases Reduces the Reinstatement of Cocaine-Seeking Behavior in Rats

**DOI:** 10.2174/157015911795017317

**Published:** 2011-03

**Authors:** Pascal Romieu, Elodie Deschatrettes, Lionel Host, Serge Gobaille, Guy Sandner, Jean Zwiller

**Affiliations:** 1 FRE 3289 CNRS, Strasbourg, France; 2 IFR37 des Neurosciences, Strasbourg, France; 3 U666 Inserm , Strasbourg, France; 4 Université de Strasbourg, Strasbourg, France

**Keywords:** Drug dependence, cocaine self-administration, cocaine-seeking behavior, epigenetic regulation, histone deacetylase, trichostatin A, phenylbutyrate.

## Abstract

Drug addiction is a chronic brain disease characterized by a persistent risk of relapse, even after a long period of abstinence. A current hypothesis states that relapse results from lasting neuroadaptations that are induced in response to repeated drug administration. The adaptations require gene expression, some of which being under the control of stable epigenetic regulations. We have previously demonstrated that pretreatment with histone deacetylase (HDAC) inhibitors reduces the cocaine reinforcing properties as well as the motivation of rats for cocaine. We show here that the same HDAC inhibitors, trichostatin A and phenylbutyrate, significantly reduced the cocaine-seeking behavior induced by the combination of a cocaine injection together with the exposure to a light cue previously associated with cocaine taking. Reinstatement of drug-seeking behavior was carried out after a 3-week withdrawal period, which came after ten daily sessions of cocaine intravenous self-administration. Our results suggest that pharmacological treatment aimed at modulating epigenetic regulation, and particularly treatment that would inhibit HDAC activity, could reduce the risk of relapse, a major drawback in the treatment of drug addiction.

## INTRODUCTION

Drug dependence is currently viewed as a chronic brain disease characterized primarily by a compulsive drug-seeking and drug-taking behavior. The development of dependence to drugs of abuse occurs over time and requires cellular adaptations. Characterization of long-term plasticity underlying addiction is currently an intensive research area and concerns mainly the characterization of changes in gene expression evoked by drugs of abuse [1]. Several studies have highlighted the importance of epigenetic regulations of gene transcription in diverse aspects of psychiatric disorders, including addiction [2-4]. Epigenetic regulations are complex mechanisms that control neuronal gene transcription by regulating the accessibility of genes to the transcriptional machinery [5]. Access to DNA in the nucleosome is achieved *via *complex associations of proteins in which post-translational modifications of histones play a major role. In general, increased histone acetylation is associated with DNA relaxation and elevated transcriptional activity, whereas decreased acetylation brought about by histone deacetylases (HDACs) results in tighter DNA coiling and gene silencing [6-8]. 

We have shown that repeated injections of cocaine regulate chromatin remodeling by inducing the methyl-CpG-binding proteins MeCP2 and MBD1 in rat brain [2]. The manipulation of chromatin remodeling enzymes by pharmacological inhibition revealed that cocaine-induced changes in chromatin structure are behaviorally relevant. We showed for instance that treatment with the HDAC inhibitors trichostatin A (TsA) or phenylbutyrate (PhB) reduces the cocaine reinforcing properties under a fixed-ratio 1 (FR1) schedule of cocaine intravenous (i.v.) self-administration protocol [9]. Experiments using the progressive-ratio schedule of reinforcement showed that HDAC inhibition also attenuated the motivation of rats for cocaine, but not for the natural reinforcer sucrose. 

A unique characteristic of addiction, from a clinical point of view, is the persistence of relapse risk long after a person has stopped taking drugs, which constitutes a major obstacle to successful treatment [10]. Much drug taking, including late relapses, follows exposure to cues previously associated with drug use [11, 12]. The cues include external sensory stimuli such as drug paraphernalia as well as interoceptive stimuli [13]. In animal models, reinstatement of drug self-administration is strongly motivated by re-exposure to even small doses of the drug, and therefore to positive reminders of drug use. Given that HDAC inhibitors were able to reduce the cocaine reinforcing properties in rats under an i.v. self-administration protocol, we investigated in the present study whether the HDAC inhibitors would also modulate the cocaine-seeking behavior reinstated by the combination of cocaine-priming and cocaine-related cues after a withdrawal period.

## MATERIALS & METHODS

### Animals and Treatment

Male Wistar rats (Janvier, France), weighing 150–175 g, were housed in standard home cages under an inverted 12 h light/dark cycle (lights on at 7:00 PM). Animals had access to food and water *ad libitum*. After surgery for i.v. catheter implantation, they were housed in individual cages. They were allowed to recover for 5–7 days before the beginning of the self-administration sessions which were conducted during the dark period. All procedures involving animal care were conducted in compliance with current laws and policies (Council directive 87848, *Service Vétérinaire de la Santé et de la Protection Animales*; permission 67-165 to JZ). Cocaine hydrochloride (Cooper, France) solution was adjusted with 0.9% NaCl to infuse an i.v. dose of 0.33 mg/kg/injection during self-administration session. TsA (Sigma-Aldrich), sodium 4-phenylbutyrate (Fluka, Sigma-Aldrich) or 10% DMSO in saline were administered i.v. Catheterization procedure into the jugular vein was performed as previously described [9]. 

### Cocaine Operant Self-Administration and Reinstatement Procedure

Studies were conducted in dark operant chambers (30 × 30 × 30 cm) located in a sound-attenuated room [9]. Each chamber was equipped with two 3 cm-diameter holes on the same wall, located 4 cm above the floor; one was selected as the active hole for delivering the reinforcer and the other as the inactive hole. They were counterbalanced between right and left position in the various groups of rats. Nose-pokes (NPs) into the inactive hole had no programmed consequence. When the number of required NPs into the active hole was reached, a 40 µl cocaine solution was delivered during a 3-sec period. A stimulus green light (3Hz flash), located 8 cm above the active hole, was paired contingently with the delivery of cocaine (3 sec). Immediately after each cocaine infusion, a white house light was switched on and persisted during a 40-sec time-out period.

For cocaine self-administration procedure [14], rats were submitted to an FR1 schedule of reinforcement during daily 1 h sessions for 10 days. In this schedule, each NP into the active hole during the valid period triggered an i.v. infusion of cocaine solution. No cutoff was applied concerning the number of self-infusions the rat was able to perform during the session. After the 10-day period of self-administration, rats were submitted to a withdrawal period of 3 weeks, during which they were housed individually in their home cages. Starting 4 days before the reinstatement session, they were i.v. injected daily with the HDAC inhibitors or with vehicle (10% DMSO in saline), the fifth and last injection occurring the day of the reinstatement, 30 min before the session. The reinstatement session started immediately after rats were given i.p. injections of 15 mg/kg cocaine. NPs performed in each hole were then recorded for 1 h. NPs in the active hole successively turned on the green light for 3 sec and the house light for 40 sec, but no cocaine solution was delivered during the reinstatement session.

## RESULTS

When rats were trained to i.v. self-administer cocaine (0.33 mg/kg/injection) during daily 1-h sessions under a FR1 schedule of reinforcement, a stable behavior could be observed after 3 sessions (data not shown), that remained until the tenth session. At the end of the self-administration period, four balanced groups of rats were established from the results obtained during the 3 last sessions, so that they displayed a similar pattern of behavior in terms of number of injections, as shown in Fig. (**1A**) as well as of NP activity in the active and inactive holes, as depicted in Fig. (**1B**). The percentage of NPs performed in the active hole by all groups was higher than 80% during the three last sessions (data not shown). Rats were then submitted to a 3-week period of withdrawal.

**Fig. (2). Effect of TsA and PhB on the combination of cocaine- and cue-induced reinstatement of cocaine-seeking behavior.**
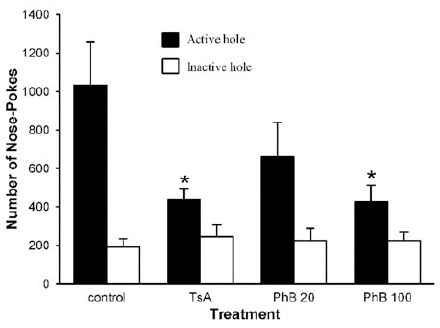
Rats were i.v. injected daily with vehicle or with the indicated HDAC inhibitor, beginning 4 days before the reinstatement session. They were treated again 30 min before being injected i.p. with 15 mg/kg cocaine and placed immediately in the self-administration chamber. Numbers of NPs, expressed as means ± sem, realized in the active and inactive holes during the 1-h test session are shown. *p < 0.05, comparison between pharmacological and vehicle treatment (One-way Kruskal-Wallis ANOVA followed by Dunn’s *post hoc* test).

Animals were treated daily with the HDAC inhibitors or vehicle for five consecutive days, the last injection occurring 30 min before the reinstatement session. Fig. (**2**) shows that during the 1-h session, i.v. injection of 0.3 mg/kg TsA significantly reduced the cocaine- and cue-induced reinstatement of cocaine-seeking behavior. When compared to control vehicle-treated animals, rats treated with TsA reduced the number of NPs directed towards the active hole by a mere 57%. Similarly, treatment with the HDAC inhibitor PhB produced a marked dose-dependent reduction in cocaine-seeking behavior in rats, since the number of NPs achieved into the active hole was reduced by 36% and 59%, when animals were treated i.v. with 20 and 100 mg/kg PhB, respectively. It is noteworthy that the NPs achieved into the inactive hole, representing about 18% of those realized into the active hole for the control group of rats, were similar in all the groups considered (Fig. **2**). 

Fig. (**3**) illustrates the cocaine-seeking behavior of the same groups of rats, but considering shorter time-intervals than the whole 1 h reinstatement session. It is shown that control rats realized most of their NPs during the first 30-min period, with a maximum occurring during the 20 to 30 min interval, and then the number of NPs progressively decreased towards the end of the session. No statistical difference could be established between the different intervals, though. In contrast, animals treated with TsA completed less NPs during the first 10-min interval, and the number of NPs remained quite stable during the entire session, achieving statistically significant differences when compared to control rats. The low dose of PhB produced a somehow linear decline in the number of NPs rats were achieving during the reinstatement session, with statistically significant differences being noticed for the second and third interval. The higher PhB dosage produced a profile similar to that observed in TsA-treated animals, with statistically significant differences, compared to control rats, being found for three out of the six 10-min time intervals considered.

## DISCUSSION

Since we have previously reported that pretreatment with HDAC inhibitors reduces the cocaine reinforcing properties as well as the motivation of rats for cocaine [9], we investigated in the present study whether these pharmacological agents were also able to affect the cocaine-seeking behavior. To this end, we trained rats under an FR1 schedule of i.v. cocaine self-administration protocol for ten days, which was followed by a 3-week withdrawal period. The reinstatement session was carried out after a treatment for 5 days with the HDAC inhibitors. A 5-day treatment was selected because we noticed previously that TsA, at the dosage of 0.3 mg/kg, produced a statistically significant effect on cocaine self-injections from the third session [9]. We show here that both TsA and PhB significantly reduced the cocaine-seeking behavior induced by the combination of a cocaine injection together with the exposure to a light cue previously associated with cocaine taking. While this does not allow to identify which one of the two parameters is primarily affected by the HDAC inhibitors, we consider that the combination of both stimuli is more appropriate to model the situation that addicted individuals are facing. While TsA had a strong effect at the dose of 0.3 mg/kg, it took up to 100 mg/kg of PhB to reduce significantly the cocaine-seeking behavior. This is actually in agreement with their respective inhibition potency: TsA, a member of the hydroxamate group of HDAC inhibitors, displays an *in vitro* IC_50_ in the nanomolar range [15], whereas PhB, from the class of aliphatic acids, has an affinity in the micromolar range [16]. The two inhibitors show no selectivity for particular members of class I, II or IV HDACs [16].

The data show that five daily sessions of TsA or PhB injection remodeled chromatin sufficiently to become behaviorally relevant, by preventing the reinstatement of drug-induced behavioral changes. This is in agreement with our previous data showing that the HDAC inhibitors reduced the reinforcing properties of cocaine [9]. The results are also in line with a recent report showing that administration of an HDAC inhibitor facilitates the extinction of cocaine-induced conditioned place preference and reduces the reactivation of place preference [17]. The similar results obtained when using the two paradigms (drug self-administration and conditioned place preference) clearly indicate that neuroadaptations taking place in response to repeated cocaine injections and underlying drug craving [18] are under the control of epigenetic regulations of gene expression, which involve modification of histone acetylation. It is noteworthy that intrahippocampal delivery of HDAC inhibitors has been shown to facilitate fear extinction in a contextual fear conditioning paradigm [19]. On the opposite, TsA administration to mice after a spatial learning task phase was found to improve the memory performances during the retention phase [20]. Together, the data suggest that other cognitive processes than only memory/learning mechanisms underlie drug extinction and drug seeking behavior, in line with the fact that drug addiction concerns other brain structures, such as the nucleus accumbens or the dorsal striatum, than those involved in spatial memory.

Since chromatin remodeling was sufficient to alter a behavior as elaborate as drug-seeking, an animal model of drug relapse, the findings provide a potential novel target for the development of treatments that would facilitate reduction in drug dependence. It should be noted that HDAC inhibitors were efficient in reducing cocaine-seeking behavior despite the severe conditions the test was carried out. In effect, one has to consider that i) we used a combination of cocaine injection and exposure to a light cue to induce cocaine reinstatement; ii) rats were not submitted to an extinction protocol after the self-administration training procedure, but to a withdrawal period. In the latter condition, being back to the context of the test chamber was probably accountable for some NP activity (in addition to drug priming) developed by the rats during the reactivation session. 

In conclusion, our results strongly suggest that treatment aimed at modulating epigenetic regulations, especially the pharmacological inhibition of HDAC activity, could reduce the risk of relapse, a major drawback in the treatment of drug addiction. 

## Figures and Tables

**Fig. (1). I.v. cocaine self-administration by rats during the 3 last FR1 sessions.**
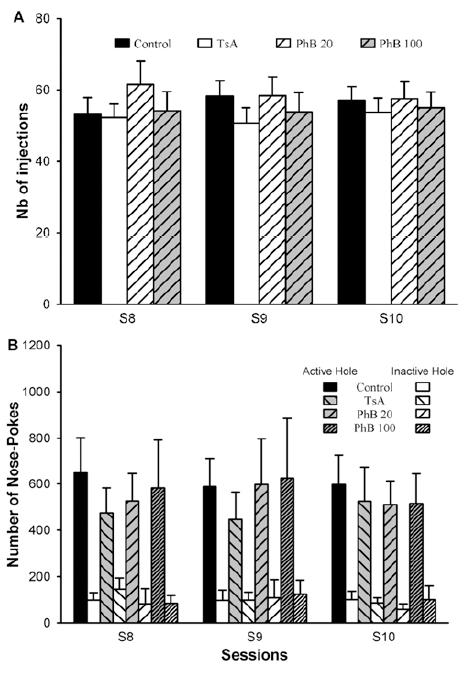
Rats were trained to self-administer cocaine (0.33 mg/kg/injection) during daily 1-h sessions under a FR1 schedule of reinforcement for 10 days. Results for the 3 last sessions are presented as (**A**), the number of self-injections performed during each session; (**B**), the number of NPs achieved in the active and inactive holes. Results are expressed as means ± sem. Rats were dispatched into several groups, as indicated, in view of the pharmacological treatment beginning 4 days before the reinstatement session. Control vehicle-treated group, n =11; 0.3 mg/kg TsA, n = 12; 20 mg/kg PhB, n = 7; 100 mg/kg PhB, n = 8.

**Fig. (3) Time-course of the effect of TsA and PhB on the combination of cocaine- and cue-induced reinstatement of cocaine-seeking behavior.**
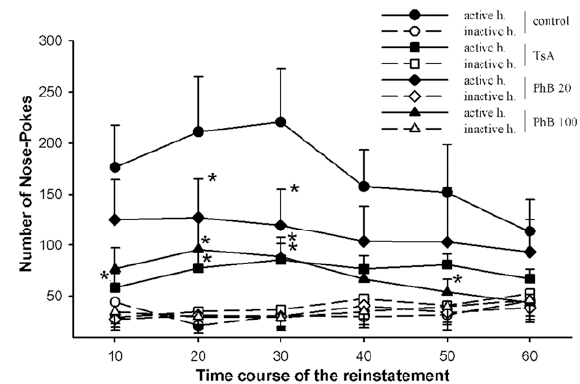
Rats were treated as indicated in the legends to Figs. (**1**) and (**2**). Numbers of NPs, expressed as means ± sem, realized in the active and inactive holes during 10-min intervals in the reinstatement session are shown. *p < 0.05, comparison between pharmacological and vehicle treatment (Two-way ANOVA, treatment X time for repeated measures, followed by the Holm-Sidak *post hoc* test).
